# Increased cognitive demands boost the spatial interference effect in bimanual pointing

**DOI:** 10.1007/s00426-016-0762-5

**Published:** 2016-03-02

**Authors:** Ioana Stanciu, Stefanie C. Biehl, Constanze Hesse

**Affiliations:** 1 School of Psychology, University of Aberdeen, King’s Campus, Aberdeen, AB24 3FX UK; 2 Clinical Psychology and Psychotherapy, Eberhard Karls University Tübingen, Tübingen, Germany

## Abstract

It is beyond controversy that in bimanual coordination tasks, parameter planning related to the movements of one hand influences the planning and execution of movements simultaneously performed with the other hand. A well-researched example of such bimanual interference is the finding that reaction times tend to be longer when preparing bimanual pointing movements with different amplitudes than for equal amplitude movements. Interestingly, these reaction time costs were found to increase when movement targets were cued symbolically (e.g., using letters) as compared to spatially. Therefore, it was suggested that interference may be primarily related to cue translation and response selection processes rather than resulting from cross-talk at the motor programming level. Here, we argue that spatial interference effects do not necessarily depend on the type of cues used but instead depend on the general task demands (difficulty). In two experiments we show that bimanual interference effects can (1) be abolished in symbolic cueing conditions when highly compatible cues placing minimal demands on response selection processes are used and (2) occur in direct/spatial cueing conditions when a secondary cognitively demanding, but movement-unrelated task is performed. Thus, our findings suggest that whether or not interference effects emerge during movement planning depends on the overall task difficulty and hence the resources available during movement preparation.

## Introduction

Even though we are able to perform with ease most everyday tasks that require asynchronous bimanual coordination, such as driving our car or ironing our shirts, experimental studies have repeatedly and consistently demonstrated that there are temporal as well as spatial coordination constraints between the two hands (for review see, Swinnen & Wenderoth, 2004). For example, Kelso, Southard, and Goodman (1979) showed that even when the two hands have to perform movements of varying difficulty and to different positions in space, participants show a strong tendency to initiate and terminate both movements at the same time (but see also, Fowler, Duck, Mosher, & Mathieson, 1991; Marteniuk, MacKenzie, & Baba, 1984). In addition to these temporal constraints, limitations to produce independent bimanual hand movements can also be observed in the spatial domain (e.g., Franz, 1997; Franz, Zelaznik, & McCabe, 1991; Kelso, Putnam, & Goodman, 1983; Spijkers & Heuer, 1995; Spijkers, Heuer, Kleinsorge, & van der Loo, 1997). For instance, Franz et al. (1991) observed spatial assimilation effects when the hands had to produce simultaneous asymmetric movements such as drawing a line with the left hand and a circle with the right hand (see also, Albert & Ivry, 2009). Furthermore, when movements are spatially incongruent (different amplitudes and/or different directions), reaction times (RTs) are usually prolonged indicating that processing times increase when the movements become more complex (Heuer, 1986; Spijkers et al., 1997). The observation that there are general RT costs for planning hand movements with asymmetric amplitudes (or directions) is often referred to as the spatial interference effect. However, there is considerable debate about the underlying mechanism(s) of this phenomenon (e.g., Blinch et al., 2014; Diedrichsen, Hazeltine, Kennerley, & Ivry, 2001; Hazeltine, Diedrichsen, Kennerley, & Ivry, 2003; Heuer & Klein, 2006; Spijkers et al., 1997).

One suggestion has been that the interference effects in bimanual coordination tasks are likely to be caused by some kind of neural cross-talk (Marteniuk et al., 1984; Spijkers & Heuer, 1995; Swinnen & Walter, 1991). More specifically, Heuer and colleagues argued that longer reaction times observed when different movement amplitudes have to be specified for the two hands (as compared to identical movement amplitudes) can be attributed to transient coupling during the movement programming phase (e.g., Heuer, 1993; Heuer, Spijkers, Kleinsorge, van der Loo, & Steglich, 1998; Spijkers et al., 1997). According to this transient cross-talk hypothesis, mutual inhibition occurs during the movement programming phase when distinct movement parameters have to be specified for the two hands simultaneously (Spijkers et al., 1997; Spijkers, Heuer, Steglich, & Kleinsorge, 2000). If participants have the opportunity to prepare their movements in advance such that movement parameter specification no longer needs to occur during the RT interval, then RTs no longer differ between symmetric and asymmetric movements (Spijkers et al., 1997). To put it simply, according to this hypothesis prolonged RTs for asymmetric movements are caused by increased processing demands during response programming. Once movement programming for both hands is finished, no further cross-talk is assumed to happen (see also Schmidt, 1975, generalised motor programing theory).

However, the hypothesis that the RT costs for asymmetric bimanual movements occur at the level of motor programming was later challenged by Diedrichsen et al. (2001). They argued that increased RTs for asymmetric movements only occur when symbolic cues are used to specify the movement targets but not when the movement targets are defined directly (spatially). In other words, in most of the initial studies on the bimanual spatial interference effect, targets were either defined by words (e.g., “short” or “long”), letters (e.g., “S” or “L”) or bars indicating the length of the movement amplitude (Heuer & Klein, 2006; Spijkers et al., 1997, 2000). Hence, to initiate the correct movements, these cues have to be identified and then translated into the required actions. In contrast, if the movement targets are presented directly such that there are only two target locations present in the workspace, no cue–response translation process is required. By comparing RTs in conditions employing either direct spatial cues or symbolic (letter) cues, Diedrichsen and colleagues could show that RT costs for asymmetric movements are limited to conditions in which the movements are cued symbolically. Based on these findings, they suggested that asymmetry costs for bimanual movements are related to response selection processes and not to increased processing demands during motor programming as initially suggested. Thus, increased RTs for asymmetric movement amplitudes are likely to be linked to the fact that two different stimulus response mapping rules have to be retrieved and applied in the incongruent (different amplitude) condition while the same mapping can be used in the congruent (same amplitude) condition (see also Albert, Weigelt, Hazeltine, & Ivry, 2007).

Interestingly, studies further investigating this suggestion came to mixed conclusions with some confirming the absence of asymmetric RT costs in direct cueing conditions (e.g., Albert et al., 2007; Diedrichsen, Ivry, Hazeltine, Kennerley, & Cohen, 2003; Hazeltine et al., 2003) and others showing that there are small but still significant costs even when movements are cued directly (e.g., Blinch et al., 2014; Blinch, Cameron, Franks, Carpenter, & Chua, 2015; Heuer & Klein, 2006). Based on this inconsistency, it was proposed that the two suggested forms of interference processes are not mutually exclusive but can occur concurrently (Diedrichsen, Grafton, Albert, Hazeltine, & Ivry, 2006; Heuer & Klein, 2006): Firstly, there are (relatively small) costs due to an increased complexity of motor programming (constraint on motor level) and secondly, there are larger costs related to increased demands of cue translation and response selection (constraint on perceptual and cognitive level; for review see Wenderoth & Weigelt, 2009). The notion that interferences during bimanual movements do not exclusively arise on a motor outflow level but are strongly mediated by cognitive factors is further supported by studies showing that RT costs for asymmetric movements are attenuated in situations in which movements are performed to identical target symbols (e.g., two circles out of circles and crosses) suggesting that selecting target positions with similar features enhances bimanual performance and eliminates RT costs for incongruent movements (Diedrichsen et al., 2003; Weigelt, Rieger, Mechsner, & Prinz, 2007; Wenderoth & Weigelt, 2009).

The phenomenon that choice RTs depend on the stimulus–response (S–R) compatibility has been studied extensively using different paradigms (Hazeltine et al., 2003; Hommel, 1997; Kornblum, Hasbroucq, & Osman, 1990; Neumann, 1990; Prinz, 1990). In short, it has been shown that response specification is generally facilitated when the similarity between stimulus and response is increased. In other words, a high compatibility between the stimulus and the required response permits a more direct parameter specification resulting in faster RTs (or reduced RT costs for incompatible movements). Hence, response selection and associated RT costs can vary strongly with the properties of the presented cues. Following this line of argument, Hazeltine et al. (2003) suggested that while the cue–response mapping in symbolic cueing conditions is highly abstract requiring a (cognitively demanding) translation of the cue into the appropriate response, direct cueing conditions place only minimal demands on the response selection process (excluded stage hypothesis).

In our study, we wanted to further investigate the claim that interference effects disappear for directly cued movements as central processes required for cue translation and response selection are bypassed. Specifically, we hypothesised that the occurrence of interference effects may be more generally linked to the task difficulty and thus the cognitive resources available for response selection and movement preparation. In the symbolic cueing conditions employed in previous studies, the cues needed to be selected, identified and subsequently translated into a motor response (applying mapping rules that needed to be retrieved from working memory). The translation of movement cues into actions, therefore, requires cognitive resources and hence may leave reduced capacity for response selection and motor programming when asymmetric movements are required. In contrast, in the direct cueing conditions, stimulus–response translation requirements—and thus cognitive demands—are negligible. If, as we propose, RT costs for asymmetric movements are linked to a limitation in central cognitive resources, they should also occur in dual-task situations in which the secondary task is completely unrelated to the movement task.

To test this idea, we asked participants to perform symmetric and asymmetric bimanual movements in two conditions in which the movements were cued directly; in one block of trials participants had to perform an additional movement-irrelevant but highly demanding attentional task shortly before or during movement preparation, while in another block no such task was required (Experiment 1). We also implemented two symbolic cueing conditions with varying cue–response compatibility mappings. In the mapping condition with high cue–response compatibility, centrally presented arrows pointed directly toward the relevant movement targets. In contrast, in the mapping condition with low cue–response compatibility, the arrows indicating the relevant movement targets were not clearly associated with the movement targets. We predicted that RT costs for asymmetric movements should arise (or at least significantly increase) in (1) the condition in which targets were directly cued and a cognitively demanding secondary task had to be performed; and (2) in conditions in which symbolic cues with low stimulus–response compatibility were implemented (requiring a demanding cue translation process). Finally, to test the generality of our cognitive resource limitation hypothesis, we conducted a second experiment testing a different secondary task. Specifically, we asked participants to execute directly cued bimanual movements while simultaneously performing a (movement unrelated) working memory task with either no-, low- or high-working memory load conditions. Generally, our findings seem to support the view that the occurrence of bimanual interference effects depends on the overall task demands.

## Experiment 1

### Methods

#### Participants

Sixteen University of Aberdeen graduate and undergraduate students (5 male, 11 female) participated in the experiment. Participants were between 21 and 30 years old (mean age 24 years), had normal or corrected-to-normal vision and were all right-handed by self-report. The experiment was approved by the ethics committee of the School of Psychology of the University of Aberdeen and written consent was obtained from each volunteer before the beginning of the experiment.

#### Setup and Stimuli

Participants sat comfortably on a height-adjustable chair in front of a table within a dimly lit room. In front of them, a 19’’ IPS computer monitor (Dell P1914S, 1280 × 1024 pixel, 30 × 37.5 cm, 60 Hz) was screwed flatly to the table surface (portrait mode) at a viewing distance of about 50 cm. A thin acrylic glass panel (30 × 37.5 × 0.3 cm) was placed on the surface of the monitor as screen protection. On the lower edge of the monitor two circular green felt-pads (1 cm in diameter) marked the starting position for the two fingers. The felt-pads were equidistant from the midpoint of the monitor edge with a distance of 9 cm between them.

**Fig. 1:**
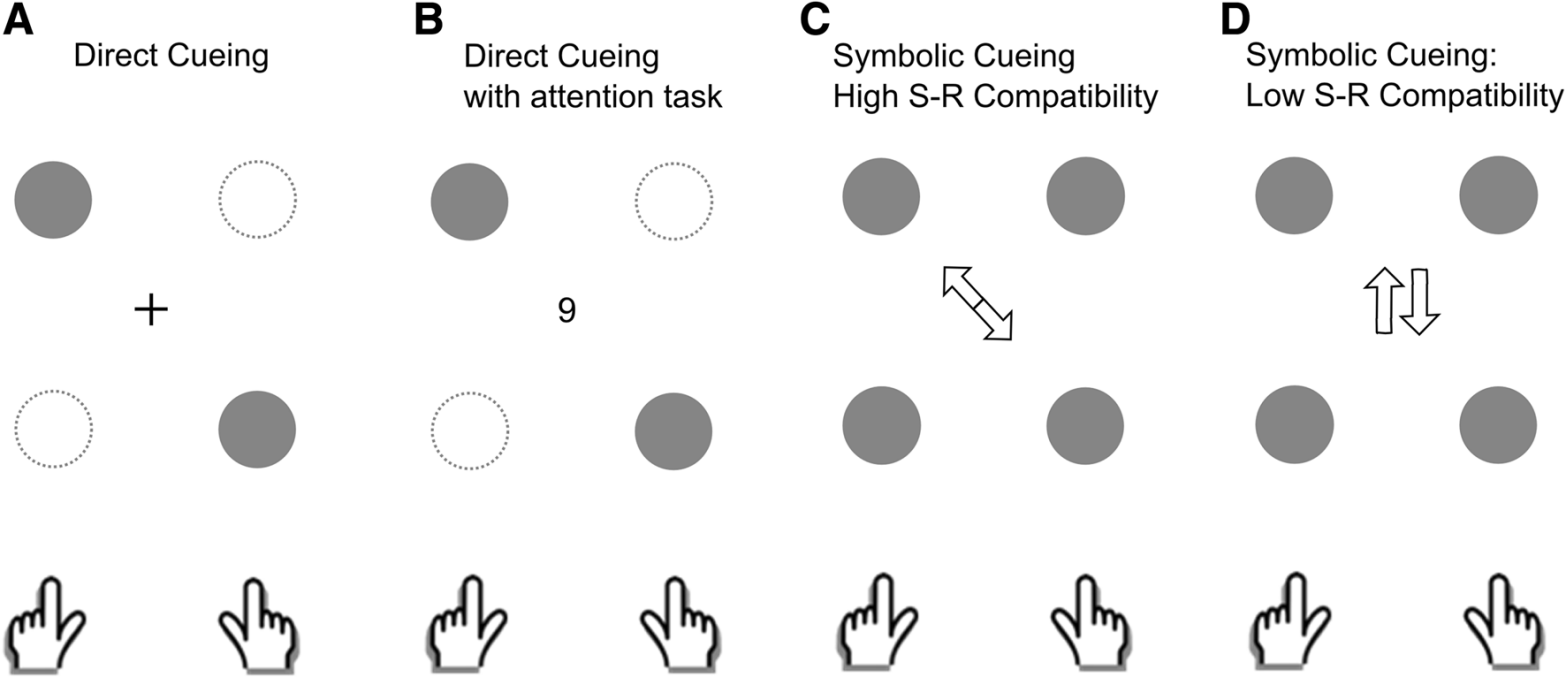
Illustration of the four different cueing conditions used in Experiment 1. Condition *A* and *B* applied a direct cueing paradigm in which participants had to move their hands to the remaining *two circles* after the preview period. In condition B, participants had to perform an additional attentional task and report a target *digit* (indicated by a *change in colour*) presented shortly before they began their movements (see “Methods” section for more information). Condition *C* and *D* employed a symbolic cueing task using *arrows* as cues whilst manipulating the stimulus–response compatibility

The targets were displayed on the monitor as red circles with a diameter of 12 mm on a grey background. There were four different possible target positions that were arranged in a rectangular fashion. The targets could appear at two different distances vertically in line with the starting positions of the left and right index fingers. The distance between the respective finger’s start position and the near target locations was 13.5 cm and the distance to the far target locations was 25.5 cm (see Fig. 1).

Pointing movements were recorded with an optoelectronic motion tracking system (Optotrak 3020, Northern Digital Incorporation, Waterloo, Ontario, Canada) at a sampling rate of 200 Hz. One light-emitting diode marker was attached to the nail of the index finger of the left and right hand, respectively. The experiment was programmed in MATLAB using the Psychophysics Toolbox (Brainard, 1997; Kleiner, 2010) and the custom-built Optotrak Toolbox (Franz, 2004). Prior to the experiment, the Optotrak was calibrated such that the Cartesian coordinate system (*x*, *y* and *z*) corresponded to the monitor plane with the origin (0, 0, 0) at the left downward edge of the monitor.

#### Procedure

At the beginning of each trial, participants placed their left and right index fingers at the start positions. Subsequently, the experimenter initiated the trial manually with a key press. The trial started with a display showing all four possible targets together with a black fixation cross in the centre between them. Participants were instructed to fixate at the fixation cross. An exception was the block in which the secondary attentional task had to be performed. In this task, the fixation cross was replaced with a centrally displayed rapid serial presentation of digits which participants were asked to attend (see below). After this preview period that lasted for 800 ms, the display changed, indicating the two targets to which participants had to simultaneously move their fingers to. Movements were parallel to the sagittal plane and always made away from the body. There were four types of movements participants could make to the remaining targets: short amplitude for both hands (SS), long amplitude for both hands (LL), left hand short amplitude and right hand long amplitude (SL), or left hand long amplitude and right hand short amplitude (LS). Depending on the task, the target positions were specified differently.

In the direct cueing condition, one target on each side was switched off after the preview period such that only one target circle was present on either side of the fixation cross. Participants had to move quickly and accurately to the remaining targets with both hands (see Fig. 1a). In the direct cueing condition with additional attentional task (see Fig. 1b), participants also had to point to the remaining two circles after the preview interval. However, either shortly before, or at the moment at which the movement targets were specified, participants had to perform an additional attentional task. Specifically, they had to attend to a rapid serial visual presentation (RSVP) stream of digits (between 1 and 9) during the trial. All digits were randomly chosen and presented for 33 ms (2 frames) with a blank interval of 66 ms (4 frames) between each presentation. All numbers were black presented on a grey background. Participants’ task was to identify a target number that was presented in white, which could randomly appear either in the first frame after 650 ms of the 800 ms lasting preview time had elapsed, or simultaneously with the movement cue. We chose two different presentation times to prevent participants from being able to predict the occurrence of the target digit during the experiment. The RSVP (only containing black numbers) continued until the end of the trial. Participants were encouraged to perform both the movement task and the visual attention task as accurately as possible.

Furthermore, we introduced two different symbolic cueing conditions both using arrows to indicate the target positions. In the high cue–response compatibility condition (see Fig. 1c) we specified the targets using white arrows (16 mm in length and touching at their ends) that pointed directly to the two target circles. After the preview period, the fixation cross was replaced by the two arrows. In this condition, participants did not need to interpret the symbols, as the cues provided direct spatial information about the targets’ locations. In contrast, in the low cue–response compatibility condition, the two arrows specifying the target positions did not point directly to the target locations but were presented next to each other pointing either straight up or down (see Fig. 1d). Note that this condition requires interpretation of the symbols and thus has the most resemblance to the symbolic cueing protocols adopted by earlier studies.

All targets remained visible throughout the trial. Once the movement targets were specified participants had 2 s to complete their movements (i.e., the position of the markers were measured for 2 s until the trial was ended). In all conditions, participants were instructed to move to the targets as quickly and accurately as possible.

The four cueing conditions were blocked and the order of blocks was counterbalanced across participants. Within each block each movement condition (short–short, long–long, long–short, short–long) was repeated 10 times resulting in a total of 40 trials per block. Before each block participants were provided with 8 practice trials to familiarise themselves with the task.

#### Data analysis

From the position signal of the Optotrak markers we calculated the resultant velocity between each frame for both markers. Movement onset was determined for each finger separately as the moment at which the resultant velocity exceeded a threshold of 0.05 m/s. Reaction time (RT) was defined as the time between the specification of the movement targets and movement onset. Similarly, movement offset was defined as the first frame at which the velocity of the markers dropped below a threshold of 0.05 m/s and the markers were less than 25 mm from the target centre in *y* direction. Movement time (MT) was defined as the time between movement onset and movement offset. Furthermore, movement accuracy was defined as the distance of the finger from the centre of the target in the *y* direction at movement offset. Movement accuracy was measured as the constant (signed) error with negative values indicating an undershoot of the target and positive values indicating an overshoot of the target, and was determined along the *y*-axis only, as this was the only dimension along which the position of the targets varied between trials.

Trials were excluded from the analysis if reaction times were shorter than 100 ms indicating movement anticipation (6 trials in total, 0.2 %) or if the lag in movement onset between the hands was larger than 100 ms (5 trials in total, 0.2 %). Reaction times were then collapsed across both hands. Furthermore, RT data were averaged across conditions with congruent movement amplitudes (SS and LL) and across conditions with incongruent movement amplitudes (LS and SL) and subsequently analysed using 4 × 2 repeated-measures ANOVAs with task (direct cueing, direct cueing with attentional task, symbolic cueing low S–R compatibility, symbolic cueing high S–R compatibility) and congruency (same or different amplitudes for both hands) as factors. Significant interactions were followed up by calculating simple main effects of congruency. Movement times were also calculated as averages across both hands but computed separately for all four movement types (i.e., congruently short, congruently long, short movements combined with long movements and long movements combined with short movements). In other words, to obtain the average movement time for short movements in the conditions in which they were combined with long movements, we averaged across the MTs obtained in the SL condition for the left hand and the movement times obtained in the LS condition for the right hand. Conversely, to obtain the movement times for long movements in the conditions in which they were combined with short movements, we averaged across the MTs obtained in the LS condition for the left hand and the movement times obtained in the SL condition for the right hand (for similar procedure see, Diedrichsen et al., 2001). The same procedure was applied to analyse the effect of movement amplitudes on movement accuracy. The data were statistically processed using a 4 (task) × 4 (movement type: SS, SL, LS, LL) repeated-measures ANOVAs. Post hoc tests were Bonferroni corrected for multiple comparisons if applicable. All values are presented as means ± SEMs. A significance level of *α* = 0.05 was used for all statistical analysis.

### Results and discussion

All participants performed above chance level in the visual attention task. On average, they reported the correct target number in 68.3 ± 4.4 % of the trials. There was a tendency for better identification performance when the digit was presented later within the preview interval (66.2 % correct after 650 ms vs. 70.4 % correct after 800 ms, *t*(15) = 2.16, *p* = 0.047, *d* = 0.54). Movement data were analysed from all trials (independent of whether the correct number was reported) as we were generally interested in the effects of sharing resources between movement preparation and an attentional task.

**Fig. 2:**
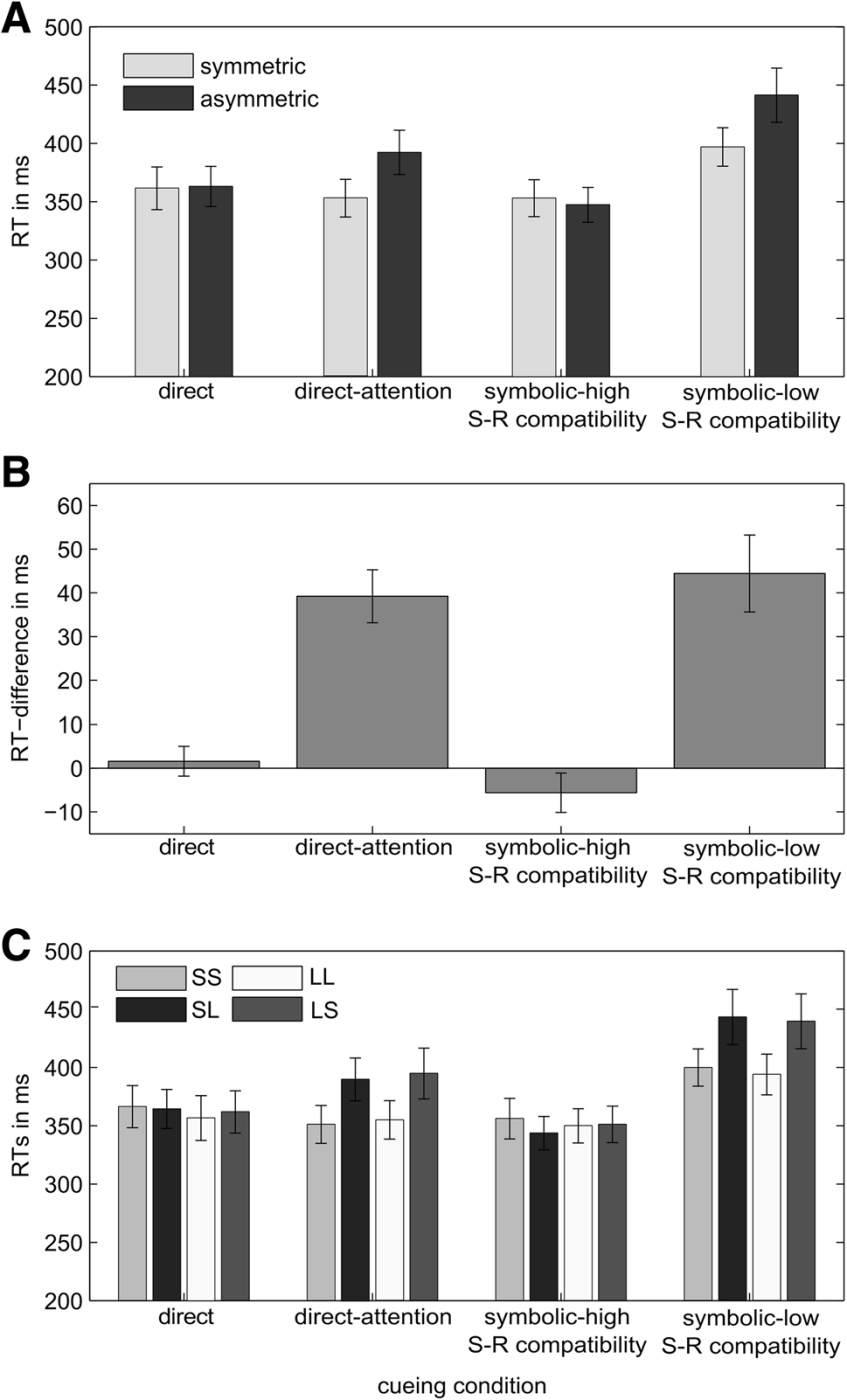
Experiment 1: **a** RTs averaged across both hands of all participants as a function of movement congruency and cueing condition. **b** Average RT difference between congruent and incongruent movements in each cueing condition. **c** RTs averaged across both hands of all participants calculated separately for all four movement types (amplitudes) and cueing conditions. *Error bars* reflect ±1 SEM between subjects

Figure 2a shows the means of the median RTs of each participant. The 4 (task: direct cueing, direct cueing with attention task, symbolic cueing low S–R compatibility, symbolic cueing high S–R compatibility) × 2 (congruency: same vs. different amplitude) repeated-measures ANOVA revealed a significant main effect of task, *F*(3,45) = 7.44, *p* = .005, $\eta_{p}^{2} = 0.33$, a significant main effect of congruency, *F*(1,15) = 29.92, *p* < .001, $\eta_{p}^{2} = 0.67$, as well as a highly significant interaction between both factors, *F*(3,45) = 21.28, *p* < .001, $\eta_{p}^{2} = 0.59$. Post hoc tests further analysing the main effect of task showed that overall RTs were slower in the symbolic cueing condition with low S–R compatibility (419 ± 20 ms) than in the direct cueing condition (363 ± 18 ms, *p* = .002) and the symbolic cueing condition with high S–R compatibility (350 ± 15 ms, *p* < .001). There was no significant difference between the RTs in the symbolic cueing condition with low S–R compatibility and the direct cueing condition with attentional task (373 ± 17 ms, *p* = .30). All other pairwise comparisons were also not significant (*p* > .81). The main effect of movement congruency cannot be meaningfully interpreted as there was a significant interaction effect between the two factors, indicating that effect of movement congruency differed between cueing conditions. To investigate how movement congruency affected RTs in the different cueing conditions, we conducted paired-samples *t* tests.

These analyses confirmed that movement congruency did not affect RTs in the direct cueing condition, *t*(15) = 0.36, *p* = .73, *d* = 0.08, and the symbolic cueing condition with high S–R compatibility, *t*(15) = 1.25, *p* = .23, *d* = 0.34. However, interestingly, in both the direct cueing condition with attentional task, *t*(15) = 6.49, *p* < .001, *d* = 1.84, and the symbolic cueing condition with low S–R compatibility, *t*(15) = 5.06, *p* < .001, *d* = 1.96, a significant movement congruency effect was found (see Fig. 2b). Hence, in line with previous research we found that performing incongruent movements results in slower RTs when symbolic (arrow) cues that place high demands on the response selection process are used but not when the movements are cued directly (e.g., Diedrichsen et al., 2001). Remarkably, however, when the movement targets were specified using arrows pointing directly to the relevant targets requiring minimal cue translation, RTs showed the same pattern as in a direct cueing task. This suggests that it is not the use of symbolic cues per se that causes movement congruency effects but that stimulus–response compatibility plays a major role (Hommel, 1997; Neumann, 1990). Furthermore, the observation that movement congruency effects occur in a direct cueing task when attention is diverted seems to indicate that not only a demanding process of cue translation but any cognitively demanding secondary task is able to elicit a movement congruency effect. Finally, it is worth pointing out that the pattern of results for congruent and incongruent movements was the same in all conditions when we analysed RTs separately for long and short movements (Fig. 2c).

As pointed out by Diedrichsen et al. (2001) the absence of RT costs for incongruent movements may possibly be a result of delayed movement programming. In other words, in certain conditions, participants may start their movements before all kinematic parameters such as movement amplitude have been fully specified (van Sonderen & van der Gon, 1991). If this is the case, then movement programming has to partly take place during movement execution which is expected to prolong the corresponding MTs in the incongruent conditions relative to the congruent conditions. In other words, if movement programming is deferred into the movement execution phase in the condition in which no movement congruency effect occurred on RTs, this would be revealed in a significant interaction effect between condition and movement type on the MT-data. However, the 4 (task) × 4 (movement type: SS, SL, LS, LL) repeated-measures ANOVA on MTs revealed no significant interaction between task and movement type, *F*(9,135) = 1.55, *p* = .14, $\eta_{p}^{2} = 0.09$, as well as no main effect of task, *F*(3,45) = 0.21, *p* = .89, $\eta_{p}^{2} = 0.01$, speaking against the deferred programming account. As expected, the analysis confirmed a main effect of movement type, *F*(3,45) = 68.94, *p* < .001, $\eta_{p}^{2} = 0.82$. It always took participants longer to perform long than short movements independent of movement congruency (all *p* < .002, see Table 1). Furthermore, it took participants significantly longer to perform a short movement in the incongruent condition in which the other hand performed a long movement than in the congruent condition (SL vs. SS; *p* < .001). Similarly, it took them shorter to perform long movements in the incongruent condition than in the congruent condition (LS vs. LL; *p* = .02) indicating accommodation effects across the two hands (e.g., Kelso et al., 1979; Marteniuk et al., 1984).

**Table 1:** Experiment 1: movement time (MT) data in ms (SEM) and movement accuracy data (Acc) in mm (SEM) for the different movement distances in each of the cueing conditions averaged across all participants (*N* = 16)

Movement distance	Cueing condition				
	Parameter	Direct	Direct attention	Symbolic, high S–R compat.	Symbolic, low S–R compat.
LL	MT	548 (31)	558 (25)	555 (26)	550 (31)
	Acc	5.4 (0.3)	5.0 (0.5)	5.8 (0.4)	5.7 (0.4)
LS	MT	545 (34)	525 (24)	539 (29)	526 (28)
	Acc	5.8 (0.4)	5.1 (0.6)	6.5 (0.8)	4.9 (0.8)
SL	MT	474 (23)	477 (21)	483 (20)	474 (23)
	Acc	7.0 (0.6)	6.5 (0.7)	6.3 (0.8)	7.3 (0.9)
SS	MT	436 (23)	435 (20)	446 (20)	432 (21)
	Acc	7.6 (0.5)	7.1 (0.5)	7.2 (0.5)	6.7 (0.3)

The first letter of the movement distance condition (L vs. S) refers to the amplitude of the hand for which the values are specified in the table, and the second letter to the amplitude of the other hand

Finally, regarding the accuracy of pointing movements in *y* direction, we analysed both: a) the average accuracy for congruent (SS, LL) movements compared to incongruent (LS, SL) movements to test if impaired planning for incongruent movements may become apparent in increased errors, and b) the average accuracy for all four movement types in all conditions (see Table 1). The latter analysis was done as it has previously been shown that participants tend to overshoot short movements if combined with long ones whilst long movements tend to remain relatively accurate, independent of the movement amplitude of the other hand (e.g., Marteniuk et al., 1984; Sherwood, 1991; Spijkers & Heuer, 1995). Regarding the effects of movement congruency on accuracy, the 4 (task) × 2 (congruency) repeated-measures ANOVA revealed no main effects of task (*p* = .37) and congruency (*p* = .56) as well as no interaction effect (*p* = .98). On average participants pointed about 6.2 ± 0.3 mm from the centre of the target circle. When determining the amplitude errors separately for all movement types, the 4 (task) × 4 (movement type: LL, LS, SL, SS) repeated-measures ANOVA revealed a significant main effect of movement type, *F*(3,45) = 4.02, *p* = .048, $\eta_{p}^{2} = 0.21$, but again no main effect of task (*p* = .36) or interaction (*p* = .13). Post hoc tests indicated that participants were less accurate in the SS condition than in the LS or LL condition (both *p* < .05) while there was no difference between the SS and SL conditions (*p* = .99). All other comparisons were also not significant (all *p* > .37). Hence, we did not find an accommodation effect for short movements when combined with long movements (as indicated by an increased overshoot) in our experiment. Possibly, this may be due to the smaller amplitude difference between the two movement options in our experiment (12 cm) compared to previous studies (e.g., 20 cm in Marteniuk et al., 1984) and/or the fact that we used discrete rather than oscillatory movements (Spijkers & Heuer, 1995).

## Experiment 2

The novel and most interesting finding of Experiment 1 is that a cognitively demanding but movement-unrelated secondary task that is performed concurrently with response selection can elicit a movement congruency effect in a direct spatial cueing paradigm. Remarkably, the size of the congruency effect observed in the dual-task situation was comparable to the size of the effect found in the symbolic cueing task with high response selection demands (39 vs. 45 ms, *p* = .57). What remains unclear, however, is which aspects of the secondary task interfered with the movement preparation process. As the resource-demanding digit identification task was completed shortly before movement onset, participants were restricted in where they could allocate their attention during the pre-movement interval. Furthermore, as soon as participants had identified the target digit, resources could potentially be freed to perform the bimanual pointing task. Therefore, we designed Experiment 2 to test if our findings would generalise to a different cognitive task which also tapped cognitive resources but a) did not manipulate attention allocation during the preview period and b) occupied resources during the whole movement preparation and execution process. Additionally, the question arises if the size of the observed RT costs for incongruent movements depends on the difficulty of the secondary task (i.e., the more demanding the cognitive task, the larger RT costs for incongruent movements). To address these issues, we replaced the perceptual (attentional) secondary task with a working memory task that required participants to retain a sequence of digits in working memory during movement preparation as well as movement execution. Moreover, we varied the amount of working memory load (no load, low load, high load) between blocks to test if RT costs were related to the difficulty of the secondary task.

### Methods

#### Participants

Nineteen University of Aberdeen graduate and undergraduate students (5 male, 14 female) participated in the experiment. One participant had to be excluded from the study as he did not follow the instructions. The remaining participants (*N* = 18) were between 20 and 32 years old (mean age 24 years) and had normal or corrected-to-normal visual acuity. One of the female participants was left-handed and the remaining participants were right-handed, as determined by self-report. The experiment was approved by the ethics committee of the School of Psychology of the University of Aberdeen and written consent was obtained from each volunteer before the beginning of the experiment.

#### Setup, stimuli and procedure

**Fig. 3:**
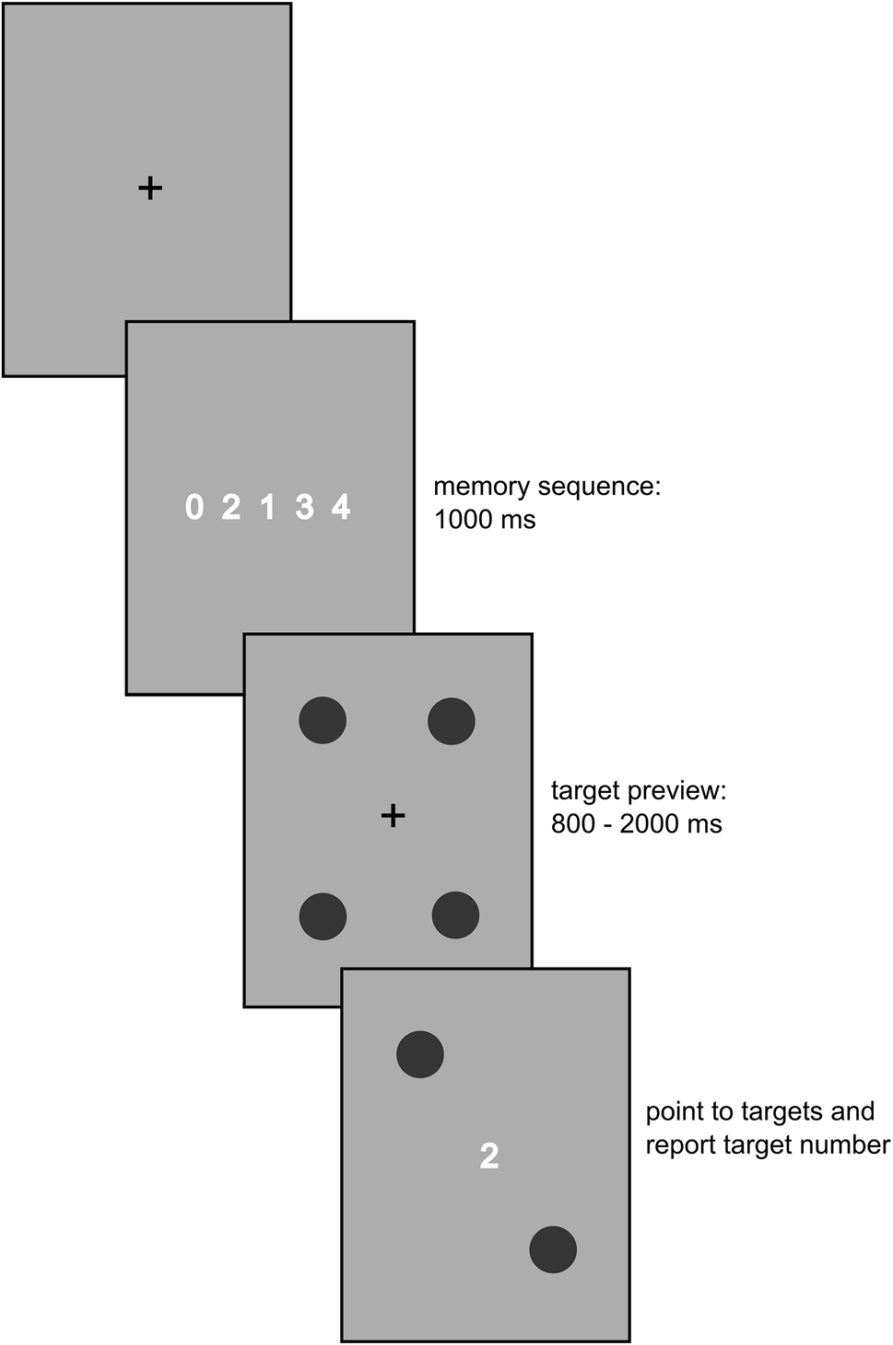
Illustration of the trial sequence in Experiment 2. The figure shows an example for a high-loadworking memory condition in which the numbers 1–4 were presented in a random sequence. Participants’ task was to report the number that followed the digit presented after a random retaining interval lasting between 800 and 2000 ms. In the depicted example the correct answer would be “1”

The setup for the experiment was identical to the one used in Experiment 1. Similarly, all movement targets and their positions were the same as in Experiment 1. However, in this experiment we only used the direct cueing condition and added a working memory task. To probe working memory, we presented, at the beginning of each trial, a sequence of five digits (between 0 and 4). The sequence differed depending on the memory task. In the low-load task the same digits were always shown in the same order: 0 1 2 3 4. In the high-load task, each memory set started with the digit zero (0) followed by the four non-zero digits (1–4) that were presented in random order (e.g., 0 3 4 2 1). Hence, participants had to remember a sequence of four digits; this procedure ensures that all digits between 1 and 4 could be used as response (see de Fockert, Rees, Frith, & Lavie, 2001 for similar procedure). Finally, in the *no*-*load task* each trial started by displaying a sequence of five zeros: 0 0 0 0 0, and there was no subsequent memory retrieval task. In all conditions, the full digit sequence was shown for 1 s (see Fig. 3). After that the memory set was removed from the screen and the four movement targets together with the fixation cross were displayed. The length of this preview period was randomly determined before each trial and could last between 800 and 2000 ms (in steps of 100 ms). After the preview, two of the movement targets were extinguished and a memory probe was presented at the position of the fixation cross (see Fig. 3). In the low-load and high-load working memory tasks, participants were requested to report the digit that followed the presented probe and to simultaneously move as quickly and accurately as possible to the two remaining movement targets. As soon as participants had started their movement (one of the index fingers had moved at least 20 mm away from the start position in *y* direction) the memory probe was removed from the screen and the fixation cross reappeared on its place. In the no-load condition, participants were instructed to ignore the probe (which was always a 0) and to not report any numbers. Furthermore, we asked participants to verbally report the probe as quickly as possible and ideally before they finished their movements. The experimenter manually entered the digit that was verbally reported by the participant after every trial. All numbers were 11 mm in size and the spaces between the numbers in the memory set were 13 mm.

The three different memory conditions were blocked and counterbalanced across participants. Each of the movement conditions (SS, LL, LS, SL) was repeated 12 times and presented in a randomised order within each block. Hence, each block consisted of 48 trials. Participants were allowed 8 practice trials before each block to familiarise themselves with the task.

#### Data analysis

All data were analysed in the same way as in Experiment 1. Again, we excluded all trials in which reaction times were shorter than 100 ms indicating movement anticipation (9 trials in total, 0.4 %) or if the lag in movement onset between the hands was larger than 100 ms (12 trials in total, 0.5 %).

### Results and discussion

Generally, participants were able to do the pointing task and the memory task simultaneously and made very few mistakes in the memory task. In the low-load condition participants reported the correct target number in 97.3 ± 1.1 % of all trials. In the high-memory load condition participants’ memory performance was slightly worse and they reported the correct number in 93.5 ± 1.6 % of all trials. However, this difference in performance between the low-load and the high-load conditions was not statistically significant, *t*(17) = 1.93, *p* = .071, *d* = 0.46. Again, we analysed the movement data from all trials independent of whether the correct response was given.

**Fig. 4:**
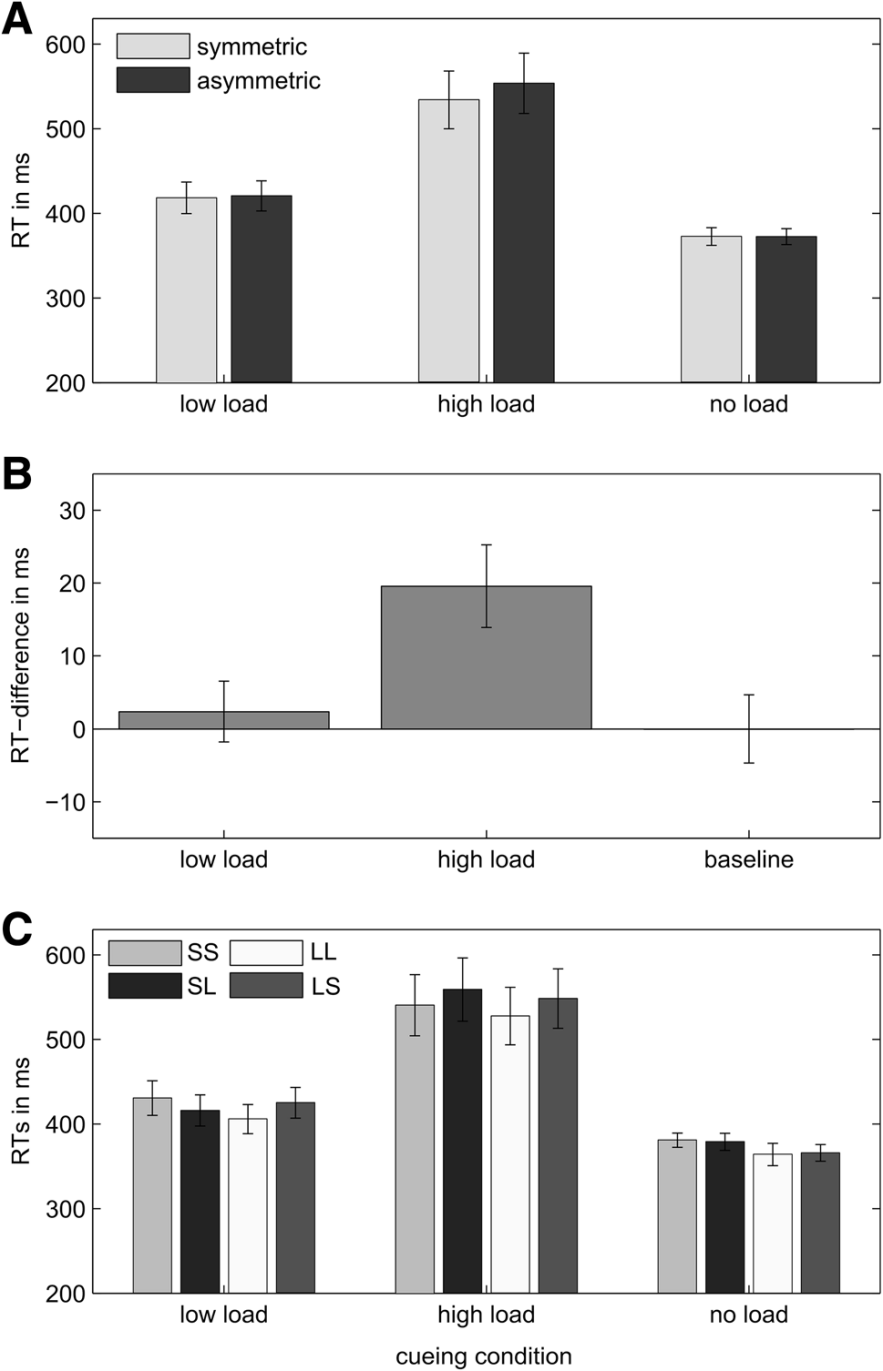
Experiment 2: **a** RTs averaged across both hands of all participants as a function of movement congruency and memory load condition. **b** Average RT difference between congruent and incongruent movements in each working memory load condition. **c** RTs averaged across both hands of all participants calculated separately for all movement types (amplitudes) in all three memory conditions. Note that movements were always cued directly. *Error bars* reflect ±1 SEM between subjects

As in Experiment 1, our main interest was in whether there was a RT difference for trials with congruent and incongruent movement amplitudes in the different working memory conditions. The RT data are shown in Fig. 4. The 3 (task: no load, low load, high load) × 2 (congruency: same vs. different amplitude) repeated-measures ANOVA showed a significant main effect of task, *F*(2,34) = 30.29, *p* < .001, $\eta_{p}^{2} = 0.64$. Post hoc analyses confirmed that RTs differed significantly between all three conditions (all *p* ≤ .003), with the no-load condition being the quickest (372 ± 10 ms), the low-load condition being slower (419 ± 18 ms) and the high-load condition being even slower by a large margin (544 ± 35 ms). Furthermore, there was a significant main effect of movement congruency, *F*(1,17) = 7.88, *p* = .012, $\eta_{p}^{2} = 0.32$. This main effect cannot be meaningfully interpreted in the presence of the significant interaction effect, *F*(2,34) = 4.29, *p* = .022, $\eta_{p}^{2} = 0.20$. To investigate how movement congruency influenced RTs in the three different tasks, we performed paired-samples *t*- tests. Movement congruency had no effect on RTs in the no-load condition (*p* = .99) and the low-load condition (*p* = .58). In the high-load condition, however, participants were significantly quicker in initiating congruent movement amplitudes as compared to incongruent ones, *t*(17) = 3.39, *p* = .009, *d* = 0.84 (Fig. 4b). Again the pattern of results for congruent and incongruent movements was consistent across conditions when RTs were analysed separately for long and short movements (Fig. 4c). Moreover, we also calculated the correlation between the percentage of correctly memorised targets in the high-load condition and the size of the interference effect across participants. A small negative, but non-significant, correlation, *r*(18) = −0.24, *p* = .17, indicates that there is a slight tendency for participants showing a larger bimanual interference effect when they found the memory task harder (less correctly named targets). Please note that overall participants performed very well (on average 93.5 % correct responses) hence the working memory task might have been too easy to detect a reliable correlation. Similarly, the lack of a congruency effect in the low-load condition can likely be attributed to the fact that, similar to the no-load condition, participants were not really required to memorise anything in this condition (apart from the traditional order of numbers). However, despite this criticism, using this task has the advantage that it controls for the possibility that merely the requirement of providing a verbal response during the movement may be sufficient to evoke a movement congruency effect. Our findings suggest that this is clearly not the case. Future studies are needed to investigate if congruency effects indeed correlate with the difficulty of the secondary task as tentatively suggested by our findings.

To check if any possible effects of movement congruency on movement programming may have been deferred into the movement execution period, we also analysed MTs. The 3 (task: no load, low load, high load) × 4 (movement type: SS, SL, LS, LL) repeated-measures ANOVA indicated that there was no significant main effect of task, *F*(2,34) = 0.36, *p* = .70, $\eta_{p}^{2} = 0.02$, as well as no interaction effect between task and movement type, *F*(6,102) = 1.09, *p* = .37, $\eta_{p}^{2} = 0.06$ (see Table 2). These findings are inconsistent with a deferred programming account. As expected there was again a significant main effect of movement type, *F*(3,51) = 144.31, *p* < .001, $\eta_{p}^{2} = 0.90$. Unsurprisingly, both long movements took significantly longer to perform than both short movements (all *p* < .001) independent of the movement distance of the other hand. Additionally, and as in Experiment 1, we found that movement times were significantly longer for short movements that were performed in the incongruent condition (SL) than for short movements performed in the congruent condition (SS) indicating an accommodation effect (*p* < .001). Similarly, we also found that long movements took shorter when they were combined with a short movement (incongruent condition) than when both hands had to move the long distance (LS vs. LL; *p* = .02).

**Table 2:** Experiment 2: movement time data (MT) in ms (SEM) and movement accuracy data (Acc) in mm (SEM) for the different movement distances in each of the memory load conditions averaged across all participants (*N* = 18)

Movement distance	Cueing condition			
	Parameter	No load	Low load	High load
LL	MT	539 (20)	551 (27)	553 (24)
	Acc	5.8 (0.8)	5.0 (1.1)	6.3 (0.6)
LS	MT	535 (21)	529 (25)	541 (23)
	Acc	5.9 (0.4)	5.8 (0.6)	3.5 (1.2)
SL	MT	474 (19)	476 (22)	482 (19)
	Acc	8.7 (0.7)	8.3 (0.8)	8.0 (0.9)
SS	MT	439 (16)	444 (22)	452 (19)
	Acc	9.2 (0.5)	8.2 (0.7)	7.8 (0.8)

The first letter of the movement distance condition (L vs. S) refers to the amplitude of the hand for which the values are specified in the table, and the second letter to the amplitude of the other hand

The 3 (task: no load, low load, high load) × 2 (congruency: same vs. different amplitude) repeated-measures ANOVA on the accuracy of the movements in vertical direction revealed neither any significant main effects (both *p* > .11) nor a significant interaction effect (*p* = .37). On average, participants tended to slightly overshoot the target with the movement endpoint being about 6.9 ± 0.4 mm away from the centre of the target in vertical direction. The data suggest again that the difference in RT between tasks cannot be attributed to a speed–accuracy trade-off. Finally, similarly as in Experiment 1, we also investigated if movement accuracy varied depending on the movement amplitude (testing for spatial accommodation effects). The 3 (task) × 4 (movement type) repeated-measures ANOVA revealed no main effects of task (*p* = .15) and no interaction effect (*p* = .32). However, like in Experiment 1, there was a main effect of movement type, *F*(3,51) = 18.05, *p* < .001, $\eta_{p}^{2} = 0.52$. Post hoc analyses confirmed that generally, participants were significantly more accurate when performing long movements than when performing short movements (all *p* < .007) independent of the amplitude of the second hand (SL vs. SS, *p* > .99 and LS vs. LL, *p* > .99). Again this is not in line with a spatial accommodation effect as reported in previous studies (Marteniuk et al., 1984; Spijkers & Heuer, 1995). The fact that the overshoot was reduced for long movement amplitudes may be related to biomechanical constraints of our setup.

## General discussion

In two studies we investigated a possible explanation for why RTs for asymmetric (or incongruent) bimanual movements are usually longer when the movements are cued symbolically but not (or to a much smaller extent) when they are cued directly. Previous studies have suggested two different, but not mutually exclusive, mechanisms that may be responsible for increased RTs for incongruent movements. Initially, interference was suggested to occur at the motor programming level as the generation of two distinct motor commands may cause mutual inhibition due to neural cross-talk during amplitude specification (Heuer, 1986, 1993; Heuer et al., 1998; Spijkers et al., 1997). However, a few years later, it was proposed that interference mostly arises at a cognitive level. According to this view, the observed RT costs for incongruent movements are attributed to the resource-demanding cue–response translation process necessary in symbolic cueing conditions (Albert et al., 2007; Diedrichsen et al., 2001, 2003; Hazeltine et al., 2003). To date, it is considered that in fact both processes may play a role in creating the bimanual congruency effect (Diedrichsen et al., 2006; Heuer & Klein, 2006). In other words, increased RTs for incongruent movements can be attributed to a small cost arising at the motor level (i.e., preference of the motor system to plan and execute symmetric movements) which occurs for both direct and symbolically cued movements and a larger cost arising at a cognitive level when cues have to be identified and translated into movement goals in the symbolic cueing conditions (for review see Wenderoth & Weigelt, 2009). Here we suggest that the overall size of the interference effect does not necessarily depend on whether or not cue identification and translation are required but depends more directly on the overall task demands.

We based our study on the view that the symbolic cueing conditions create a dual-task situation. In other words, in addition to movement programming and execution, participants have to identify the cues, retrieve and select the correct stimulus–response mapping rules (keeping the associated mapping rules in working memory) and subsequently select the appropriate motor responses. In other words, symbolic cueing requires participants to develop internal codes for each movement and associate these with the presented symbolic cues (such as letters, bars or words). Hence, the process of cue translation requires cognitive resources and may therefore leave less capacity for the relevant processes related to response selection and motor preparation (see also, Albert et al., 2007; Hazeltine et al., 2003). In contrast, in the direct cueing conditions, no resource-demanding cue–response translation process is required as there is a direct mapping between the stimulus and the required response. Hence, we hypothesised that RT costs for asymmetric movements vary with the difficulty of the secondary task and may be relatively independent of whether or not this task is movement related. We tested this prediction in Experiment 1 in two ways: firstly, we introduced two different symbolic cueing conditions that varied the compatibility between the presented cue and the required response. In line with our prediction, we found asymmetry costs for movements performed in the symbolic condition with low cue–response compatibility (i.e., high translational load) but not in conditions with high cue–response compatibility (i.e., low translational load). Secondly, we tested whether bimanual interference occurs in direct cueing conditions when participants perform a secondary cognitively demanding, but movement unrelated, task. Interestingly, we found RT costs for asymmetric movements in the dual-task condition suggesting that any kind of dual-tasking coinciding with response selection and action preparation may be sufficient to evoke a movement congruency effect. This finding makes it unlikely that interference effects observed in previous studies are a direct consequence of cue translation and corresponding response selection processes but can instead, more generally, be attributed to increased cognitive demands in symbolic cueing tasks. In other words, interference effects in bimanual actions may only become apparent in more complex (difficult) movement tasks.

To further confirm this notion, we conducted a second experiment in which we introduced a different secondary task that varied the amount of working memory load during movement preparation and execution. In line with our hypothesis that RT costs for asymmetric movements vary with the cognitive task demands, we found longer RTs for incongruent movements when the working memory load was high. However, even though the RT costs occurred reliably, they were overall smaller for the working memory task than for the dual-task condition in Experiment 1 (about 40 ms in Experiment 1 vs. 20 ms in Experiment 2). There are a couple of possible reasons for this discrepancy. On the one hand, the reduced RT costs in Experiment 2 may reflect that a mere working memory task requires less resources than a task comprising a combination of visual attention and working memory components as used in Experiment 1 (note that participants had to keep the identified number in working memory until the end of the trial). On the other hand, the secondary task in Experiment 2 may just have been simpler than the task used in Experiment 1. Tentative support for this suggestion comes from the finding that the amount of correctly reported target numbers was much higher in Experiment 2 (Exp. 2: 93.5 % vs. Exp.1: 69.8 %).

Before discussing the implications of our study we need to address one important methodological difference to many previous studies employing a direct cueing paradigm (e.g., Albert et al., 2007; Diedrichsen et al., 2001; Hazeltine et al., 2003). In these studies, direct cues were presented as a sudden onset within the visual field (i.e., the two targets to which participants have to move their hands appeared). In contrast, in our study, we presented all four possible target locations during the preview period (similar to the symbolic cueing conditions) and defined the targets by a visual offset of the non-target locations (for a similar procedure see also, Blinch et al., 2014). We chose this procedure as it was pointed out by Hazeltine and colleagues (2003) that many studies investigating differences between symbolic and direct cueing conditions (e.g., Diedrichsen et al., 2001; Hazeltine et al., 2003) displayed all relevant movement targets before the movement was required in the symbolic cueing conditions but not in the direct cueing conditions. Hence, partial movement pre-programming may have taken place in the symbolic cueing conditions before cue presentation. Adjusting these pre-planned movement programs after cue presentation may in turn have induced the observed cross-talk in these conditions. By always displaying all possible movement targets in both the direct and the symbolic cueing conditions during the preview period, this potential confound is avoided. Finally, we think that it is unlikely that this procedure substantially changes our findings, compared to studies using target onsets, as it has been shown that (when attention is unfocused as in the current study) visual onsets and offsets are equally effective in attracting attention to different locations in space (Theeuwes, 1991).

Overall, this is the first study that indicates that the occurrence of the bimanual interference effect does not merely depend on the type of cues used (symbolic vs. direct) but rather seems to be related to the general cognitive demands the task poses. In other words, even when a secondary task that is completely unrelated to the movement task is performed, interference effects can be observed. Notably, these findings may partly resolve the debate of why interference effects have consistently been found in symbolic cueing conditions but rarely (and to a much smaller extent) in spatial cueing conditions. It is important to point out that the link between bimanual movement studies and dual-task performance was originally suggested by Hazeltine and colleagues (2003). However, our findings that bimanual interference can (1) be abolished in symbolic cueing tasks by minimising the response requirements and (2) be created in direct cueing conditions by maximising processing demands provides the first convincing empirical evidence for the notion that bimanual interference effects are primarily related to dual-task demands and overall task difficulty.

Regarding the question of how our account relates to the previous notion that interference occurs at two stages, i.e., during motor programming and cue translation, we think that it has the advantage that it can explain previously observed effects without assuming two different and independent underlying processes. Specifically, our results may help to understand why some, but not all, studies found bimanual interference effects in direct cueing tasks. For instance, Blinch et al. (2014) found RT costs when participants performed directly cued asymmetric movements without visibility of their hands using a handheld stylus. It stands to reason that it is a much more demanding task to perform movements with a tool and without visual feedback than it is to point directly with both fingers while having both hands fully visible. Consequently, the task is likely to require more attentional resources yielding the observed congruency effect. Similarly, movement congruency effects were found to be larger in direct cueing conditions when reversal movements rather than discrete pointing movements were investigated (e.g., Heuer & Klein, 2006) suggesting again that movement complexity may affect the size of the observed interference effect. However, even though our suggestions seem to fit nicely with some of the findings from previous studies there are also instances in which a simple explanation in terms of task demands is not instantly obvious. For example, in a recent paper, Blinch et al. (2015) reported small but significant interference effects (12 ms) in a relatively simple direct cueing task employing discrete pointing movements. One methodological difference to previous studies was, however, that target buttons were used as movement goals requiring participants to perform relatively accurate movements which are potentially more resource demanding (Hesse & Deubel, 2010). In other words, we suggest that factors that relate to movement difficulty (such as endpoint accuracy, target visibility and movement speed) may determine the amount of bimanual interference measured in different paradigms.

However, we also need to point out that our findings cannot provide a definite answer on the question at which exact processing stages the interference effect arises. Clearly the current findings can be reconciled with the proposition that interference occurs at a motor level as increasing the task demands in a direct cueing task may leave less capacity for movement programming thereby enabling transient coupling to occur. On the other hand, engaging in a movement-related (e.g., cue translation) or movement-unrelated (e.g., attentional) secondary task also leaves less resources for stimulus identification and response selection thereby allowing interference effects to emerge.

Finally, our finding that RTs are prolonged when a movement-unrelated cognitively demanding task has to be performed indicates that movement planning relies on the same central resources as needed for the execution of conscious perceptual tasks. This is in line with previous studies on unimanual reaching and grasping movements showing that movement preparation is less efficient (as indicated by longer RTs) when resources have to be shared between concurrent perceptual and visuomotor tasks (Hesse & Deubel, 2011; Hesse, Schenk, & Deubel, 2012; Kunde, Landgraf, Paelecke, & Kiesel, 2007; Similä & McIntosh, 2015). Therefore, our study provides further evidence against the view that perception and action processes may be controlled by separate attentional mechanisms allowing for efficient task sharing between visuomotor and perceptual processes without dual-task costs (Enns & Liu, 2009; Norman, 2002).

In conclusion, we showed that RT costs for incongruent bimanual movements do not depend on whether the movements are cued symbolically or directly, but on the overall processing demands of the task at hand. The harder the task, the more likely it is that dual-task interferences become apparent, suggesting that perceptual/cognitive and visuomotor tasks compete for the same limited resources.
